# “Most tantumising state of affairs”: Mathematical and non-mathematical in quantum-like understanding of thinking

**DOI:** 10.3389/fpsyg.2022.934776

**Published:** 2022-09-12

**Authors:** Arkady Plotnitsky

**Affiliations:** Literature, Theory, Cultural Studies Program and Philosophy and Literature Program, Purdue University, West Lafayette, IN, United States

**Keywords:** quantum-like theories and models, consciousness, the unconscious, reality-without-realism, ideality-without-idealism

## Abstract

This article addresses the effectiveness of the predictive modeling of cognition and behavior based on quantum principles and some of the reasons for this effectiveness. It also aims, however, to explore the limitations of *mathematical* modeling so based, quantum-like (Q-L) modeling, and all mathematical modeling, including classical-like (C-L), in considering human cognition and behavior. It will discuss certain alternative approaches to both, essentially philosophical in nature, although sometimes found in literary works, approaches that, while not quantitative, may help compensate for limitations of mathematical modeling there. Most Q-L and C-L approaches beyond physics are realist, insofar as they offer representations of human thinking by the formalism of quantum or classical physical theories. The position adopted in this article is based on the non-realist assumption that such a representation *may not be possible*, which is not the same as that *it is impossible*. I designate interpretations that do not make this assumption reality-without-realism, RWR, interpretations, and in considering mental processes as ideality-without-idealism, IWI, interpretations.

## Introduction

This article addresses the effectiveness of the predictive modeling of cognition and behavior based on quantum principles and some of the reasons for this effectiveness. It also aims, however, to explore the limitations of *mathematical* modeling so based, also known as quantum-like (Q-L) modeling, and ultimately all mathematical modeling, including classical-like (C-L), based in the mathematics of classical physics, in considering human cognition and behavior. It will discuss certain alternative approaches to both, essentially philosophical in nature, although sometimes found in literary works. These approaches may help to compensate for the limitations of mathematical modeling in cognitive and social sciences, and be used together with mathematical modeling there, in contrast to physics, where mathematical modeling is more or less sufficient.^1^ I shall only discuss Q-L models or theories based in quantum mechanics (QM) (as are most Q-L models) and quantum field theory (QFT), two currently standard forms of quantum theory, in respectively low and high energy regimes. (Alternative quantum theories such as Bohmian mechanics, will be put aside.) As explained in Section “Quantum theory: Physical postulates, mathematical formalism, and reality without realism,” the mathematical model adopted by a theory will refer here to the mathematical formalism of this theory, while a theory to the overall assemblage of concepts comprising it.

Ironically, the limitations of Q-L mathematical modeling in cognitive and social sciences in part arise from some of the same fundamental *epistemological* principles that ground this modeling and quantum theory itself, at least in certain (non-realist) interpretations, designated here as “reality-without-realism” (RWR) type interpretations. The effectiveness of mathematics in physics famously compelled E. Wigner speak of “the unreasonable effectiveness of mathematics in natural sciences,” although he made an important qualification, discussed later, to the effect that mathematics can only describe but not account for initial conditions of the phenomena considered (Wigner, 1960, p. 14). Mathematics does not appear to be as effective in cognitive and social sciences, as Wigner was undoubtedly aware, as he must have been of its more limited effectiveness in natural sciences other than physics.^2^ That we do have C-L and Q-L mathematical models in cognitive and social sciences may be more surprising, given that classical and quantum physics, as does relativity, mathematically idealize natural phenomena by disregarding most of their aspects perceived by human subjects. C-L and Q-L models, too, often use this idealization, the limitations of which are more apparent if one considers the situation in informational terms.

The data considered in physics is a form of information, which can be treated mathematically as Shannon information (a collection of bits). Shannon information is based on disregarding the semantic content of information, in accordance with information theory, either classical, which deals with information processing by means of classical physical systems, or quantum, which deals with information processing by means of quantum systems. The latter information qua information is still classical, but it cannot be created by using classical systems and obeys different principles of processing. Shannon information is still obtained in the experience of human subjects and is communicated, by means of language (along with mathematical and technical terminology) to other human subjects. This communication must be unambiguous to conform to the requirement of physics as a mathematical-experimental science of natural phenomena. Along with using a (mathematized) physical theory in assigning probabilities to the events considered, we must share their verification for this theory to be such a science. Science is a human enterprise and as such inevitably involves extra-scientific elements. However, sharing information is human, too. Science, including physics, capitalizes on this aspect of human experience and the possibility that this communication may be made unambiguous, not the least by using mathematics.

While cognitive and social sciences are still sciences, the information they consider is not restricted to Shannon information, which complicates the use of mathematics. This appears to be especially the case when using either C-L or Q-L models in dealing with cognition or thinking (a more general concept primarily used in this article). Cognitive sciences do allow for the use of mathematics, in particular probability theory, even in dealing with information that is not strictly Shannon information. The history of mathematical modeling there has been long dominated by C-L probabilistic or statistical models, borrowed from classical statistical physics or, sometimes, chaos and complexity theories. During the last decades, however, Q-like models became more prominent in these fields, as in other fields outside physics. The mathematical formalism of quantum theory is, first, inherently probabilistic because only probabilistic predictions are, in general, possible on experimental grounds, and second, it uses a different (non-additive) type of probability calculus than classical physics does. While *physically described* by classical physics, the experimental data obtained in quantum experiments *cannot be predicted* by classical physics. This incapacity led to the rise of quantum theory, from M. Planck’s discovery of the black body radiation law in 1900 on, eventually leading to QM and QFT. Beginning with A. Tversky and D. Kahneman’s pioneering work in the 1970–1980s (e.g., Tversky and Kahneman, 1973, 1983), it has been the presence of probabilistic data akin to those of quantum physics that suggested using Q-L models (e.g., Haven and Khrennikov, 2013; Busemeyer and Bruza, 2014).

The first question that this article asks is what are the ontological and epistemological reasons for using Q-L models, vs. C-L ones? This question is not merely philosophical, because it also concerns the scientific understanding of the phenomena considered by such models or theories, just as in quantum theory, QM or QFT, and its interpretations. Although our thinking is commonly, including here, assumed by Q-L models to be arising due to the neurological workings of the brain, such models need not be assumed, and are not assumed here, to arise from the quantum physics of the brain. A Q-L model may apply even if the physics of the brain is assumed to be classical. This physics, whether classical or quantum, will be disregarded here, as it is in many Q-L approaches. There are (hypothetical) theories that assume consciousness or thinking to be an effect of the quantum physics of the brain, such as, prominently, those by R. Penrose, beginning with (Penrose, 1995). How the physics of the brain makes thinking or consciousness, as we experience it, remains an unanswered question, sometimes referred to as “the hard problem of consciousness” (Chalmers, 2010). It appears to be designated as the hard problem of *consciousness* because our manifested inner experience is that of consciousness, and not that of the unconscious, *inferred* from our conscious thinking. The unconscious is, however, important to my argument here. While assumed to be responsible for mental processes, the brain will be treated as, *physically*, a “black box,” relating the informational input and output, encountered from either the inside or the outside of a human subject.

This type of move was made by S. Freud (who started his scientific career as a neuroscientist and initially wanted to approach thinking neurologically) in establishing psychoanalysis. It was pursued by him as a *scientific* project, as a science of the mind, analytically decoupled from the functioning of the brain, assumed responsible for “mental life” (e.g., Freud, 1969a,b). This aim of his project can be ascertained, regardless of how one views his assumptions or his success in achieving this aim. It is a more complex question whether, the mind or part of it, especially the unconscious, is a *mental* black box in the sense of the impossibility of accounting for how the mind, for example, as a Q-L informational system, produces such outputs. This impossibility may also place new limits on using mathematical information theory, classical or quantum, in this case. While decoupling the mind from the brain, Freud, aimed at accounting, through the unconscious, for the workings of the mind, by providing a mental ontology of these workings. Some Q-L approaches also aim to do so, although the mental ontologies they consider are different from that of Freud, especially by virtue of their mathematical nature. Mathematics played no role in Freud’s representation of human thinking, which was conceptual and narrative, with that of the Oedipal complex as its most famous and most controversial concept and narrative.

Whether a representation of the reality considered, or of all this reality, is possible even in quantum theory, defined by Shannon information, is the main philosophical question asked by this article. It argues that one need not assume that it always is. Specifically, in certain interpretations, designated here as reality-without-realism (RWR) interpretations, beginning with that of N. Bohr, the ultimate nature of the reality responsible for quantum phenomena is placed beyond representation or even conception. On the other hand, (observed) quantum phenomena are viewed as representable, in fact by classical physics. Understood more generally, this view defines the philosophical position of this article as the reality-without-realism, RWR, view, or when dealing with thinking, the ideality-without-idealism, IWI, view.

The article considers the reasons why the RWR/IWI view is possible, even if not necessary, in considering Q-L models of human thinking, while, again, also arguing for the limitations of these models, and ultimately any mathematical models of human thinking. Many of these reasons are, unsurprisingly, parallel to those that are led to the RWR view in considering quantum phenomena and QM or QFT, making the corresponding phenomena in human science *epistemologically* Q-L, insofar as one adopts the RWR/IWI view. At the same time, Q-L phenomena need to be understood in their own terms.

## Quantum theory: Physical postulates, mathematical formalism, and reality without realism

This section considers the physical, mathematical, and epistemological architecture of quantum theory, and why this architecture *allows* for RWR-type interpretations, one of which, defined by certain additional assumptions, is adopted by this article. Do quantum phenomena or quantum theory *require* such interpretations? It would be difficult to argue such a case, and it is not my aim to do so. I begin with the mathematics of quantum theory, which gave rise to Q-L models beyond physics.

Since the publication of J. von Neumann’s classic book (Von Neumann, 1932), QM and QFT commonly use Hilbert-space mathematical formalism. There are other versions, some more abstract ones, such as those of C*-algebras or category theory, all of which are (more or less) equivalent mathematically. Hilbert-space formalism remains dominant, however, as it is in Q-L models. I outline the key features of this formalism and using it, restricting myself to QM for now (QFT involves further complexities, discussed later):

(1) QM uses Hilbert-spaces, over complex numbers, ℂ–abstract vector spaces of any dimension, finite or infinite, which possess the structure of an inner product that allows lengths and angles to be measured, as in an n-dimensional Euclidean space (a Hilbert space over real numbers, ℝ);
(2) The feature of the formalism especially important in QM is the *non-commutativity* of the Hilbert-space operators, known as “observables,” which are *mathematical* entities associated, in terms of probabilistic predictions, with *physically* observable quantities by using (3);
(3) One needs Born’s rule or an analogous rule (such as von Neumann’s projection postulate), which establishes the relation between the so-called “quantum amplitudes,” associated with complex Hilbert-space vectors, and probabilities as real numbers, by using square moduli of amplitude (technically, these amplitudes are first linked to probability densities);
(4) The probabilities involved are non-additive: the joint probability of two or more mutually exclusive alternatives in which an event might occur is not equal to the sum of the probabilities for each alternative, as in classical probability theory, but obeys the law of the addition of “amplitudes” for these alternatives, to the sum of which Born’s rule is then applied.

I shall, for convenience, refer to Born’s rule from now on. Also, von Neumann’s postulate is more conveniently connected to ontological representations of the independent quantum processes, as defined by von Neumann’s unitary evolution, representations precluded by RWR-type interpretations. In the simplest case, when ψ is a wave function for a point particle in the position (Hilbert) space, Born’s rule tells us that the probability density function *p (x, y, z*) for predicting a measurement of the position at time *t*_1_ is equal to |ψ(*x*,*y*,*z*,*t*_1_|^2^. Integrating over this density gives the probability or (if one repeats the experiment many times) statistics of finding the particle in a given area.^3^ Although Born’s or related rules arise naturally from the formalism, they are added to rather than are contained in it. We do not know why they work, but they do.

Some history might be helpful for understanding the physical and epistemological principles underlying the formalism and its use in RWR-type interpretations. The reason is that, unlike von-Neumann’s axiomatic formulation of an existing theory, which can be interpretated in one way or another, the invention of QM by Heisenberg (initially not using the Hilbert space formalism) was grounded in these principles. They were his starting point. According to Bohr, writing in the wake of Heisenberg’s discovery:

“In contrast to ordinary mechanics, *the new quantum mechanics does not deal with a space–time description of the motion of atomic particles*” (Bohr, 1987, v. 1, p. 48; emphasis added).

An RWR-type epistemology was, thus, at the origin of QM. The mathematical features of the formalism were inferred (surmised and sometimes guessed), by Heisenberg from the physical features of quantum phenomena, as grounded in the RWR view, and the principles or postulates reflecting these features:

(1) The postulate of quantum discreteness, the QD postulate, according to which all observable quantum phenomena are discrete relative to each other (which is different from the atomic discreteness of quantum objects);

(2) The postulate of the probabilistic or statistical nature of quantum predictions, the QP/QS postulate, maintained, in contrast to classical physics, even in considering individual quantum objects, no matter how elemental, and accompanied by the non-additive character of quantum probability and rules, such as Born’s rule (a version of which was used by Heisenberg); and

(3) The correspondence *postulate*, based in Bohr’s correspondence *principle*, which, as understood by Bohr, required that the predictions of quantum theory must coincide with those of classical mechanics in the classical limit, but was given by Heisenberg a mathematical form, postulating that the equations and variables of QM convert into those of classical mechanics in the classical limit.

Given that Heisenberg’s derivation of QM (Heisenberg, 1925/1968), which depended on (3), is not my concern here, I shall only mention (3) in passing (I have considered his derivation on several previous occasions) (Plotnitsky, 2021c) (pp. 101–144). (1) and (2) are more fundamental. Implicit in Heisenberg’s approach, was another postulate, designated here as the quantum individuality (QI) postulate, which assumes that each quantum phenomenon is individual and unrepeatable, unique. All these postulates, except for (3), apply in Q-L models of human thinking and decision making, at least in the present (RWR-type) view.

One of the key points in Heisenberg’s derivation was that “in order to complete the description of radiation [in conformity, by the correspondence principle, with the classical Fourier representation] it is necessary to have not only frequencies but also the amplitudes” (Heisenberg, 1925/1968) (p. 263). The equations of QM must, then, formally contain amplitudes as well as frequencies. These amplitudes could, however, no longer be classical physical functions over ℝ(as part of a continuous representation of motion) and were instead related to transitions, *always discrete*, between quantum phenomena, observed in measuring instruments impacted by quantum objects. They were formal mathematical entities over ℂ, “probability (density) amplitudes,” linked, *via* Born’s rule, to the probabilities of outcomes of quantum experiments. The probability amplitude is ⟨λ_*i*_|ψ⟩ (λ_*i*_ is an eigen value and ψ is the wave function), and the corresponding probability is P_i_ = ⟨λ_*i*_|ψ⟩2. In this view, quantum amplitudes and linear superpositions do not represent anything physical, unlike in classical physics where both represent physical processes. They are only part of the mathematical machinery that enables quantum predictions, at least in an RWR-type interpretation, implicitly assumed by Heisenberg at the time of his discovery.

Consider the polarization of a photon. There are two possible outcomes of measurement: for example, the horizontal state *H* and the vertical state *V*. In the RWR view, however, one would not say that before it is measured the photon is in a superposition of two physical states. The wave function allowing one to predict either physical state *H* or *V* is written as |ψ⟩=α|*h*⟩+β|*v*⟩ with the probability amplitudes of |ψ⟩ associated with state vector |*h*⟩ given by α and |*v*⟩ given by β. In a random experiment, the probability of the photon, when its polarization will be measured, to be horizontally polarized is|α|2 and vertically polarized is |β|2 (by Born’s rule). That, however, need not, and in the RWR view does not, mean that |ψ⟩=α|*h*⟩+β|*v*⟩ represents the photon in a superposition of two *physical* states, *H* and *V*, as nothing can be said about what happens between observations in the RWR view. Only the (mathematical) state vectors, designated |*h*⟩ and |*v*⟩ are in a linear superposition, with given amplitudes, and not photons.

Heisenberg’s “scheme” contained another crucial feature: “What I really like in this scheme is that one can really reduce all interactions between atoms and the external world… to transition probabilities” (Heisenberg, Letter to Kronig, 5 June 1925; cited in Mehra and Rechenberg, 2001, v. 2, p. 242). QM was only predicting the effects of these interactions, observed in measuring instruments, and not the behavior of quantum objects. This view was adopted by Bohr and became a defining feature of his interpretation, *in all its versions*, eventually leading Bohr to his concept of phenomenon, defined by what is observed in measuring instruments in quantum experiments, and his ultimate, RWR-type, interpretation (Bohr, 1987, v. 2, p. 64). Bohr changed his interpretation a few times. This requires one to specify to which version of his interpretation one refers, which I shall do as necessary, while focusing on his ultimate interpretation (in the present interpretation). The designation “the Copenhagen interpretation” requires even more qualifications as concerns whose interpretation it is and at what point in time. Hence, I avoid this designation entirely.

By quantum theory I refer to a set of conceptual schemes accounting for quantum phenomena.^4^ Such schemes are clustered into specific theories, two of which, currently standard, are discussed here: QM (in low-energy, non-relativistic, regimes) and QFT (in high-energy, relativistic, regimes). The history of any theory is accompanied by the history of its interpretations, defined by concepts added to a theory, such as those that establish how the theory relates to the phenomena it considers. I define a *mathematical* model as a set of mathematical structures that enable such relations. As that of a theory, the concept of a mathematical model has a long history and diverse definitions (e.g., Frigg and Hartmann, 2012). The present concept of a mathematical model is, however, sufficient to accommodate the models that I consider. The history of QM has been shaped by a seemingly uncontainable proliferation of interpretations. It is not possible to survey them here. Even each rubric on the standard list (the Copenhagen, the many-worlds, consistent-histories, modal, and so forth), contains different versions. The case is only a bit less prohibitive in QFT.

I consider next the concept of reality-without-realism (RWR) and the RWR view, which grounds the present and related interpretations of quantum theory, such as that of Bohr, as well the present interpretation of Q-L theories and models. I have discussed this concept previously, most extensively in Plotnitsky (2021c). It is grounded in more general concepts of reality and existence, assumed to be primitive concepts and not given analytical definitions. By “reality” I refer to that which is assumed to exist, without making any claims concerning the *character* of this existence or reality, which claims define realism. By contrast, the absence of such claims allows one to place this character beyond representation or even conception, as in the RWR view. I understand existence as a capacity to have effects on the world with which we interact. The assumption that something is real is made, by inference, from such effects. Accordingly, the RWR view assumes, as do most realist view, that nature, as matter, exists independently of thought, which implies that it had existed when we did not exist and will continue to exist when we will no longer exist. There are exceptions to this view, such as, famously, that of Bishop Berkeley, that deny the existence of nature or anything apart from thought. The idea is useful in suggesting that any conception of how anything exists, or even that it exists, belongs to thought. It need not follow, however, that something which such concepts represent or to which they relate otherwise, for example, by placing it beyond representation or conception, does not exist.

Realist thinking is manifested in the corresponding theories, commonly representational in character. Such theories aim to represent the reality they consider, in modern, post Galilean, physics by mathematical models, idealizing this reality. It is possible to aim, including in quantum theory, for a strictly mathematical representation of this reality apart from physical concepts, at least as they are ordinarily understood, say, in classical physics or relativity. It is also possible to assume an independent architecture of the reality considered, while admitting that it is either (A) not possible to represent this architecture or (B) even to form a rigorously specified concept of it, either at a given moment in history or even ever. Under (A), a theory that is merely predictive could be accepted for lack of a realist alternative, usually with the hope that a future theory will do better by being a representational theory. What grounds realism most fundamentally is the assumption that the ultimate constitution of reality possesses properties and the relationships between them, or, as in (ontic) structural realism, just a structure, the constituents of which are not defined in terms of properties (Ladyman, 2016). Such properties, relationships, or structures may either be ideally represented or known, or be unrepresentable or unknown or even unknowable, but still conceivable, usually with a hope that they will eventually be represented. The assumption of this constitution as at least conceivable, even if not knowable, is arguably the weakest, Kantian, form of realism (Kant, 1997) (p. 115). Most realist theories in physics and beyond are representational.^5^

Thus, classical mechanics (used in dealing with individual objects and small systems), classical statistical mechanics (used in dealing, statistically, with large classical systems), chaos theory (used in dealing with classical systems that exhibit a highly non-linear behavior), or relativity, special and general, are realist theories. While classical statistical mechanics does not represent the overall behavior of the systems considered because their mechanical complexity prevents such a representation, it assumes that the individual constituents of these systems are represented by classical mechanics. In chaos theory, which, too, deals with systems consisting of large numbers of atoms, one assumes a mathematical representation of the behavior of these systems. Relativity poses insurmountable difficulties for our general phenomenal intuition because the relativistic law of addition of velocities (defined by the Lorentz transformation) in special relativity,$s=\frac{v\,u}{1\,{\left(v\,u/c\right)}^{2}}$, for collinear motion (*c* is the speed of light in a vacuum), is beyond any possible intuitive conception. Relativity, however, special and general, still offers a mathematically idealized representation of the physical reality it considers. All theories just mentioned are based on the assumption, workable in them, that one can observe physical phenomena without disturbing them, and as a result, identify them with the corresponding objects and their independent behavior, and (ideally) represent this behavior and predict it by using this representation.

The RWR view is, by contrast, grounded in the assumption that observable effects of physical reality entail a representation of these effects but not a representation or even a conception of how these effects come about. Such a representation or conception may not be possible, and it is not in the RWR view, in the case of the ultimate constitution of physical reality responsible for quantum phenomena. I shall speak of the *weak* RWR view when this reality is only beyond representation, and the *strong* RWR view when it is beyond conception. The concept of RWR can apply in mental domains, for example, in mathematics or psychology, and it will be so applied here, under the additional rubric of ideality without idealism (IWI). An RWR/IWI-type theory or interpretation, thus, assumes different levels of *idealizations* of reality, some allowing for a representation or conception and other not.

That the epistemological cost of the RWR view, especially, the strong RWR view, is often seen as exorbitant. This is is not surprising, especially given the discontent, equally common, even with QM or QFT as such in view of its irreducibly probabilistic nature and its resistance to realist interpretations. In addition to this philosophical discontent, based in the realist imperative, guiding A. Einstein and others, quantum phenomena defied many assumptions concerning the workings of nature and thought previously considered as basic. The way in which they do so was manifested in many famous quantum experiments, beginning with the double-slit experiment and extending to many others, showing equally strange or (under classical assumptions) paradoxical phenomena.^6^ These assumptions have served us for as long as human life itself, in part arising due to the neurological constitution of our brain, which and, thus, our thinking have evolutionarily developed in our interaction with objects consisting of billions of atoms, in dealing with which classical-like representations are effective or even evolution-wise necessary. More accurately, these representations are something that classical physics mathematically refines, as emphasized by Bohr and Heisenberg (e.g., Heisenberg, 1930, pp. 11, 64–65; Bohr, 1987, v. 2, pp. 68–69). There is, however, no special reason to assume that our thinking should be able to represent or even conceive of how nature works at very small scales. As noted, while respected by classical physics, these assumptions were already challenged by relativity, even as a realist theory. This suggests the primacy of the philosophical attitudes in Einstein’s and others’ “skepticism about the necessity of going [as far as the RWR view] in renouncing customary demand as regards the explanation of natural phenomena” (Bohr, 1987, v. 2, p. 63).

Bohr grounded his interpretation (in all its versions) in the irreducible role of measuring instruments in defining quantum phenomena and, in the ultimate version of his interpretation, in the strong RWR view, which placed quantum object beyond conception. The behavior of the observable parts of measuring instruments, defining quantum phenomena, was idealized as representable, by means of classical physics. Measuring instruments were, however, also assumed to have quantum strata through which they interact with quantum objects. Eventually, Bohr adopted the term “phenomenon” to refer strictly to what is observed in measuring instruments, as effects of this interaction (Bohr, 1987, v. 2, p. 64). The ultimate constitution of the reality responsible for quantum phenomena was associated with quantum objects. The interpretation adopted here takes a more stratified view, following (Plotnitsky, 2021a,c). The ultimate RWR reality responsible for quantum phenomena is an idealization assumed to *exist independently of our interactions with it and thus of observation*. By contrast, the concept of a quantum object is an idealization that, while still of the RWR-type, only applies *at the time observation*, defined by the interactions between the ultimate RWR-type reality and the instrument.

This view will be transferred here to our understanding of human experience as unique at any point of time in any observation from the inside or the outside. In the case of human experience, there are no entities analogous to quantum objects, such as electrons or photons, whose physical identity could be maintained in quantum experiments. The observed *states* of quantum objects are different, too. However, while mental representations can have *parts* shared between different subjects, they are ultimately different for each subject. In quantum physics, a measured physical state of, say, an electron is always that of an electron. One of the crucial aspects of quantum physics is that particles within the same type (electrons, photons, and so forth) are indistinguishable. In high-energy (QFT) quantum regimes, unlike low-energy (QM) regimes, one can in one and the same experiment register different particles, which support the present view of quantum objects as an idealization only applicable at the time of observations. One, however, still deals within a finite set of particles, with each type defined by a finite set of observable effects, associated with such quantities as mass, charge, or spin. By contrast, human thinking presents us with an uncontainable manifold of unique effects, only few of which are the same.

Two key concepts defining classical physics and relativity, (classical) “causality” and (classical) “measurement,” become no longer applicable in QM in RWR-type interpretations. By “classical causality” I refer to the claim that the state, X, of a system is determined, in accordance with a law, at all future moments of time once it is determined at a given moment of time, by the state A, and A is determined by the same law by any of the system’s previous states. This assumption implies a concept of reality (which defines this law), making this concept of causality ontological. There are several reasons for my choice of “classical causality,” rather than just causality, used more commonly. The main one is that it is possible to introduce alternative, probabilistic, concepts of causality, applicable in QM or QFT, including in RWR-type interpretations, where classical causality does not apply (e.g., Plotnitsky, 2021c, pp. 207–218).

Some, beginning with P. S. Laplace, have used “determinism” to designate classical causality. I define “determinism” as an epistemological category referring to the possibility of predicting the outcomes of classically causal processes ideally exactly. In classical mechanics, when dealing with individual or small systems, these two concepts become equivalent. On the other hand, classical statistical mechanics or chaos theory are classically causal but not deterministic in view of the complexity of the systems considered, which limit us to probabilistic or statistical predictions concerning their behavior. In quantum phenomena, deterministic predictions are, in general, not possible even in considering the most elementary quantum systems. This is, as stated, because the repetition of identically prepared quantum experiments in general leads to different recordings of the observed data, and unlike in classical physics, this difference cannot be diminished beyond the limit, defined by *h*, by improving the capacity of measuring instruments. Hence, the probabilistic or statistical character of quantum predictions holds in interpretations of QM or QFT or alternative theories (such as Bohmian mechanics) that are classically causal. QM or QFT are not classically causal in (strong) RWR-type interpretations because the ultimate nature of reality responsible for quantum phenomena is assumed to be beyond conception. (Classical causality would imply at least a partial conception of this reality.) This leads to a different nature for the recourse to probability in quantum theory in RWR-type interpretations. According to Bohr:

[I]t is most important to realize that the recourse to probability laws under such circumstances is essentially different in aim from the familiar application of statistical considerations as practical means of accounting for the properties of mechanical systems of great structural complexity [in classical physics]. In fact, in quantum physics we are presented not with intricacies of this kind, but with the inability of the classical frame of concepts to comprise the peculiar feature of indivisibility, or “individuality,” characterizing the elementary processes (Bohr, 1987, v. 2, p. 34).

The “invisibility” refers to the indivisibility of phenomena in Bohr’s sense, defined by what is observed in measuring instruments and the impossibility of considering quantum objects independently of their interactions with these instruments. “Individuality” refers to the assumption, designated here as quantum individuality (QI) postulate, according to which each quantum phenomena is unique—individual and unrepeatable. Some interpretations of QM, such as those by Von Neumann (1932) and Dirac (1958) assume classically causal views of the behavior of quantum objects, with probability or statistics brought in by measurement. That, however, was not Bohr’s view, at least after he abandoned his Como argument (Plotnitsky, 2016, pp. 122–125; Plotnitsky, 2021c, pp. 67–68). “The classical frame of concepts” may appear to refer to the concepts of classical physics, and it does include them. By the time of this statement (in 1949), however, Bohr adopted the strong RWR view, which gives the phrase “the classical frame of concepts” a broader meaning: All representational concepts are classical. The concepts of classical physics are (mathematized) refinements of concepts formed by our phenomenal thinking, as a product of our neurological machinery. This refinement may, however, not be available for representing the ultimate constitution of reality responsible for quantum phenomena or the ultimate constitution of nature in general. As noted, this was no longer the case in relativity, which was, however, able to invent physical concepts beyond human intuition to represent the reality considered. As an RWR-type interpretation, Bohr’s or the present interpretation, makes no claims concerning the world, and hence does not assume the world to be probabilistic any more than classically causal. It only makes claims concerning our interactions with the world, which are irreducibly probabilistic in dealing with quantum phenomena. The world, apart from the human world, has no probabilities, and makes no measurements or predictions; only we do, and quantum physics helped us to realize this fact (Plotnitsky, 2016, 2021c).^7^

The concept of quantum measurement adopted in this article is no longer that assumed in classical physics (or relativity) either. It follows (assuming in addition that the concept of a quantum object only applies at the time of an observation) Bohr’s view, leading him to his concept of “phenomenon,” as referring strictly to what is observed in measuring instruments. The term “measurement” is a remnant of classical physics or still earlier history, beginning with ancient Greek thinking and the rise of geometry, geo-*metry*. In Bohr’s or the present view, a quantum measurement *does not measure* any property of this reality, which it would be assumed to possess before or even during an observation. An act of observation is the establishment, *creation*, of a quantum phenomenon, new each time, by an *interaction* between an instrument and a quantum object. Then what is so observed can be measured classically.

I shall now explain Bohr’s concept of complementarity. Defined most generally, complementarity is characterized by:

(A) A mutual exclusivity of certain phenomena, entities, or conceptions; and yet
(B) The possibility of considering each one of them separately at any given point; and
(C) The necessity of considering all of them at different moments of time for a comprehensive account of the totality of phenomena that one must consider in quantum physics.

In classical mechanics, we can comprehend all the information about each object within a single picture because the interference of measurement can be neglected. This allows us to identify the phenomenon observed with the object under investigation and establish the quantities defining this information, such as the position and the momentum of the object, in the same experiment. In quantum physics, this interference cannot be neglected and leads to different experimental conditions for each measurement and their complementarity, in correspondence with the uncertainty relations, as reflected in the non-commutative nature of the corresponding operators in the formalism.^8^ The situation implies two incompatible pictures of what is observed, as phenomena, in measuring instruments. Hence, the *possible information* about a quantum object, the information *to be found* in measuring instruments, could only be exhausted by the mutually incompatible evidence obtained under different experimental conditions (Bohr, 1987, v. 2, p. 40). On the other hand, once made, either measurement will provide the *complete actual information*, as complete as possible, at this moment in time. One could never obtain the complementary information at the same time, because to do so one would need simultaneously to perform a complementary experiment, which is not possible.

One gains further insight into quantum measurement by considering the so-called “cut.” According to Bohr:

This necessity of discriminating in each experimental arrangement between those parts of the physical system considered which are to be treated as measuring instruments and those which constitute the objects under investigation may indeed be said to form a *principal distinction between classical and quantum-mechanical description of physical phenomena*. It is true that the place within each measuring procedure where this discrimination is made is in both cases largely a matter of convenience. While, however, in classical physics the distinction between object and measuring agencies does not entail any difference in the character of the description of the phenomena concerned, its fundamental importance in quantum theory … has its root in the indispensable use of classical concepts in the interpretation of all proper measurements, even though the classical theories do not suffice in accounting for the new types of regularities with which we are concerned in atomic physics (Bohr, 1935, p. 701).

There are two common misunderstandings of this and related statements by Bohr. First, Bohr’s statement may suggest that, while observable parts of measuring instruments are described by means of classical physics, the independent behavior of quantum objects is described by means of the quantum-mechanical formalism. This type of view has been adopted by some, for example, Von Neumann (1932) and Dirac (1958), moreover, under the assumption of the classical causal independent behavior of quantum objects, with probability brought in by measurement. It was not, however, Bohr’s view, at least after the Como lecture (Bohr, 1987, v. 1, pp. 52–91; Plotnitsky, 2016, pp. 122–125; Plotnitsky, 2021c, pp. 67–68). Bohr only says that the observable parts of measuring instruments are described by means of classical physics and that classical theories cannot account for quantum phenomena. But he does not say that the independent behavior of quantum objects is represented by the formalism of QM. His statement only implies that quantum objects cannot be treated classically. Bohr’s insistence on the indispensability of classical physical concepts in considering measuring instruments is often misunderstood as well. According to Bohr, the classical description can and, to enable communicable accounts of experiments, must apply to the *observable* parts of measuring instruments. The instruments, however, also have a quantum stratum, through which they interact with quantum objects, which interaction would not be possible otherwise. This interaction is quantum and hence, it cannot be observed and, in RWR-type interpretations, represented or even conceived of.

The situation under discussion is commonly referred to as the arbitrariness of the cut or the “Heisenberg-von-Neumann cut,” because the term [*Schnitt*] was favored by Heisenberg and von Neumann. As Bohr noted, however, while “it is true that the place within each measuring procedure where this discrimination [between the object and the measuring instrument] is made is … largely a matter of convenience,” it is true only largely, but not completely. This is because “in each experimental arrangement and measuring procedure we have only a free choice of this place within a region where the quantum-mechanical description of the process concerned is effectively equivalent with the classical description” (Bohr, 1935, p. 701). Thus, the ultimate constitution of the physical reality responsible for quantum phenomena, observed in measuring instruments, is never on the measurement side of the cut. Neither are the quantum strata of the instruments through which the latter interact with this reality.

If, however, a quantum object is only defined, as in the present view, at the time measurement, rather than as something that exists independently, one might ask: Could one still speak of the same quantum object, say, the same electron, in two or more successive measurements? Do these measurements register the same electron? Rigorously speaking, under this assumption, a prediction based on a given measurement and the measurement based in this prediction could only concern a new electron, and not the electron that we assumed to exist in the first measurement. To consider them as the same electron is, however, a permissible idealization in low-energy (QM) regimes. This idealization is still statistical because a detection of the original electron in the second measurement is not guaranteed. On the other hand, speaking of the same electron in successive measurements in high-energy (QFT) regimes is meaningless, because they can register quantum objects of different types, say, an electron in the first and a positron or photon in the second, which also means that if the second measurement registers an electron, the electron registered in the first measurement could have changed into something else in between before a new electron was born (Plotnitsky, 2016; Plotnitsky, 2021c, pp. 279–292). QFT, thus, justifies the present concept of a quantum object and the three-partite architecture defined by the ultimate constitution of the reality responsible for quantum phenomena (a reality assumed to exist independently), quantum objects (as an idealization applicable at the time of observation), and quantum phenomena.

Nothing be said or even thought concerning what happens between experiments in RWR-type interpretation. According to Heisenberg:

There is no description of what *happens* to the system between the initial observation and the next measurement. …The demand to “describe what happens” in the quantum-theoretical process between two successive observations is a contradiction in adjecto, since the word “describe” refers to the use of classical concepts, while these concepts cannot be applied in the space between the observations; they can only be applied at the points of observation (Heisenberg, 1962, pp. 57, 145).

The same would apply to the word “happen” or “system,” or any word we use, whatever concept it may designate, including reality, although when “reality” refers to that of the RWR-type, it is a word without a concept attached to it. As Heisenberg adds: “But the problems of language are really serious. We wish to speak in some way about the structure of the atoms and not only about ‘facts’—the latter being, for instance, the black spots on a photographic plate or the water droplets in a cloud chamber. However, we cannot speak about the atoms in ordinary language” (Heisenberg, 1962, pp. 178–179). Nor is it possible in terms of ordinary concepts, from which ordinary language is indissociable, or, in the RWR view, even in terms of physical or mathematical concepts.

Heisenberg’s formulation allows for a *mathematical representation* of this reality apart from physical concepts, at least as the latter are ordinarily understood, as in classical physics or relativity (e.g., Heisenberg, 1962, pp. 145, 167–186). This view was indeed adopted by Heisenberg at the time of this statement, although not at the time of his discovery of QM. The words “happens” or “physical” (or any word) need no longer be part of this representation, only mathematical symbols are, even if their use is described with the help of ordinary language. That does not affect the possibility of a mathematical representation of the ultimate constitution of the reality responsible for quantum phenomena between observation, because this use need not imply that they pertain to physical reality. Thus, saying that a wave function represents this reality need not imply that this reality is represented in in terms of ordinary or physical concepts, and as noted, there is no special reason to assume that they will be able to describe nature on the atomic scale. One might indeed doubt that one can do so mathematically, given that mathematics, too, is human, a product of our evolutionary development. We might be just fortunate to be able to use it physics, including quantum physics, as Heisenberg observed (Heisenberg, 1930, p. 11).

I close this section on the quantum individuality (QI) postulate in conjunction with the “no cloning” postulate, both (suitably adjusted) crucial to human thinking. The QI postulate states that every quantum phenomenon, and thus every quantum experiment is strictly individual and unrepeatable, given that, as Bohr said, “in general, one and the same experimental arrangement may yield different recordings” (Bohr, 1987, v. 2, p. 73). That refers to both recordings defining a given experiment, with the first providing the initial data and the second used to verify predictions based on these data. Both recordings will be different either if we repeat the whole procedure in the same set of experimental arrangements, or if we build a copy of the apparatus and will set it up in the same. This is always possible because both copies of the apparatus could be controlled classically. By contrast, their interaction with quantum objects cannot be controlled. Bohr refers to “the finite and uncontrollable interaction between the object and the measuring instruments in the field of quantum theory” (Bohr, 1935, p. 700). The statistics of multiple experiments performed in the same experimental settings will be the same. On the other hand, an individual quantum experiment, either the initial state of the observed part of the apparatus or in the outcome of the experiment cannot be reproduced with the same outcome, as it is always possible, in principle, in classical physics, because the interference of measurement can be neglected. All data in quantum experiments is classical and can be communicated, but it cannot, in any individual case, be recreated by a different experiment.

This impossibility is embodied in the no-cloning postulate, correlative to the QI postulate. As do other forms of the no cloning postulate in interpretations of QM, the present form of the postulate embody the no-cloning theorem in the formalism of QM. The theorem forbids the creation of identical copies of an arbitrary unknown quantum state: that is, a quantum system prepared in a state |ϕ⟩ unknown to an observer and the information it potentially defines (as classical information obtainable for means of this system) cannot be copied. Hence, no unitary universal cloning machine exists that would clone arbitrary unknown quantum states, in contrast to the universal Turing machine, which is classical.^9^ As a technical finding based in the formalism of QM, the no cloning theorem is open to interpretations as concerns its physical meaning, and its derivations are based on epistemological assumptions, implicit as they may be. The form of the no-cloning postulate adopted here is in accord with strong RWR-type interpretations. Less radical epistemological assumptions, for example, a weak RWR-type interpretation, or even some forms of realism, may also allow one to form no cloning postulates, grounded in the no cloning theorem.

## Is human thinking quantum-like, and in what sense?: Qualia and the quantum-like

An observation of a human subject by an outside agent (such as another human subject) could register representational entities, such as statements, that could be treated as identical for certain purposes. This treatment is akin to the way identical quantum objects, such as electrons, could be treated in quantum theory, although, in the present view, the identity is only a permissible idealization of the unique nature of each quantum phenomenon and the ultimate constitution of reality responsible for it. To this reality, unlike phenomena, we have, in the RWR view, no access, just as we don’t from the outside to human thinking, even conscious thinking. We can, however, access our conscious thinking more fully (even if still not completely) from the inside. Ultimately, this is the only thing which we can access. The existence of everything else is an inference made by thinking of the basis of our phenomenal experience, possibly accompanied by a claim concerning a representation of what is so inferred, or lack thereof, by a phenomenon. Just as in quantum physics, in each instance of human thinking, one deals with the unique and unrepeatable, and in the RWR view, inaccessible, state of the ultimate reality that may have observable (classical) informational effects, except that as defined by qualia (qualitative phenomenal properties), the information concerning human thinking is not containable by Shannon information. Qualia, classical in the first place, cannot be idealized as identical in the way quantum objects can.

Accordingly, while the irreducible role of observation in defining the phenomena considered, and the QI, QD, QP/QS, and no-cloning postulates could be maintained in theorizing, in a Q-L and RWR way, human thinking, there are differences in how these postulates work. Physical objects of whatever kind, classical or, as just noted, quantum, do not, at least in the present view, have consciousness or the unconscious, no inner experience. It’s been suggested, for example, in some so-called panpsychist approaches to the hard problem, that material objects and hence quantum objects do possess something akin to consciousness, even if in a much lesser degree than humans or animals do. Such views will be put aside. Consciousness and thinking are assumed here to be strictly human (or possibly found in other animals), as products of our evolutionary biological and specifically neurological development.

The uniqueness of each inner experience and the discreteness of each phenomenon, if “observed” (that is, inferred to one or another degree) based on some evidence, from outside, are represented in QI and QD postulates, which also imply the no cloning postulate. When possible, our estimates concerning any future evidence are unavoidably probabilistic or statistical, in accordance with the QP/QS postulate. In this epistemological sense, human thinking is Q-L. As such, it may be seen in terms of RWR/IWI type reality, if considered from the outside. It may be assumed to allow for an inner mental ontology (while retaining all four postulates), combining conscious and unconscious parts, and containing Q-L (as RWR-type) strata and C-L strata. This ontology is partially accessible, as inner ontology, in its classical aspects, from the outside or more fully from the inside. There is no comparable knowledge that would allow ontological assumptions concerning the ultimate constitution of the reality responsible for quantum phenomena (Such assumptions can of course be made on other grounds).

This situation is, thus, related to the hard problem of consciousness or thinking. The hard problem is that of explaining the nature of our conscious experience as the experience of qualia. It does not appear to be possible to explain it in functional physicalist terms. The functionalist approach only allows one to handle a limited range of problems related to consciousness, “easy problems,” but not for this experience itself, which is “the hard problem:” “When it comes to conscious experiences, this sort of explanation [by functions] fails. What makes the hard problem hard and almost unique is that it goes *beyond* problems about performance of functions” (Chalmers, 2010, p. 8).

Chalmers uses the idea of a “zombie” to illustrate the difference between experiences and functioning: a zombie is something indistinguishable from a human being in terms of its behavior but has no inner experience. We don’t know if zombies, especially (which is crucial) zombies that possess *all aspects* of manifested human behavior, exist, although Chalmers appears to think that they might. Related questions arise in considering digital AI, at least as concerns those “behaviors” of computers that we associate with the effects of thinking. The other side of this problematic is the question whether computers can think in the way humans do, a question that has been debated equally intensely. The argument that they do not shaped Penrose’s physicalist approach to the physically quantum nature of consciousness and thinking. His argument was in part grounded in Gödel’s theorems, the first of which tells us that there are unprovable mathematical propositions that may be true, from which Penrose inferred that human mathematicians do not think like (classical or even quantum) digital computers “think,” if the latter think at all (Penrose, 1995, pp. 105–112). This type of argument may, however, apply if one does not take a physicalist view. One can extend this problematic to other forms of AI, such as a biologically based one (represented by so called “androids” in science fiction). A “reverse” question is whether it is possible to create artificial beings that could do *all* that human beings can without in fact being human, for example, without having emotions or dreams, and hence the unconscious. This question was suggested by P. Dick’s in his (1968) science-fiction novel, *Do Android Dream of Electric Sheep?* (Dick, 2008), the title of which invokes dreams, a qualia-defined phenomenon, and in the later (1982) movie based on it, *Blade Runner*, the title referring to the occupation of the main protagonist (who may be an android).

While one must give Chalmers due credit for his contribution, the hard problem is not new. Among the obvious precursors are M. Proust, in literature, on whom I comment below, and Freud, who stated the hard problem, if without naming it.^10^ According to Freud:

We know two kinds of things about what we call our psyche (or mental life): firstly, its bodily organs and scene of action, the brain (or nervous system) and, on the other hand, our acts of consciousness, which are immediate data and cannot be further explained by any sort of [functional scientific] description. Everything that lies between is unknown to us, and the data do not include any direct [functional] relations between these terminal points of our knowledge [about mental life]. If it existed, it would at the most afford an exact localization of the processes of consciousness and would give us no help toward understanding them” (Freud, 1969a, p. 14).

Acts of consciousness or thinking, including the unconscious (inferred from some acts of consciousness), remains beyond the reach of functional treatment, as it was at the time of Freud, thus leaving the hard problem in place, an immense progress in neuroscience since then notwithstanding. As noted, Freud’s theory was epistemologically C-L, by virtue of the representational (realist) nature and classically causal view of mental life, considered, as it was, independently of their biological emergence. On the other hand, the RWR, or in this case, IWI view allows one to rethink thinking as experience, as defined by two forms of reality, Q-L and C-L, by analogy with quantum theory (in RWR-type interpretations):

(1) The ultimate constitution of reality responsible for all events of thinking, is Q-L reality, assumed here to be an RWR or IWI type reality (even though this reality may ultimately be material rather than mental, which need not require a physicalist view); and

(2) All *representations* to our mind and hence all qualia, conscious or unconscious, as effects of this reality, are classical.

Such representations can only be predicted from the outside (or even from inside) in probabilistic terms. The RWR/IWU reality responsible for these representations cannot be accessed even from the inside, let alone from the outside [from which one can access only a limits portion of reality (2)], and hence cannot be copied. Nor, just as in the case of quantum phenomena, can the classical-like information content, contained in these representations, be copied.

Memory is classical, both short term and long term, with the latter creating a vast “archive” of classical representations, from which each recollection extracts only a small portion, although any recollection may be a product, effect, of the RWR or IWI-type efficacity. It need not follow, however, that, while we have no awareness of this efficacity (we only have awareness of its representational and hence classical effects), this efficacity is available to our unconscious representations. It might instead be seen as equally productive of both conscious and unconscious C-L representations, with long term-memory archiving the unconscious ones. The question is whether the ultimate nature of the unconscious, or something of which even the unconscious is only an effect, is of a RWR/IWI type. There are also conscious effects of temporal continuity. This continuity may, however, only be apparent, constructed by the brain, and is underlain by the discreteness of unconscious events. The unconscious is not limited to creation of *small* fluctuation-type effects defining consciousness, as shown by our dreams or long-term memory, which can discontinuously switch us between distant moments in time. This makes it difficult to attribute to the unconscious the continuous temporality of conscious experience, defined by the sequence of past, present, and future “now” instants (e.g., Derrida, 1976, p. 67).

This scheme is in accord with the argument of this article concerning the double structure of measuring instruments as both classical in their observable parts and quantum in those parts of them through which they interact with quantum objects, or in the present view, the ultimate nature of physical reality responsible for both quantum objects and quantum phenomena at the time of observation. As Q-L, the efficacy of the experiences of consciousness would be parallel to this quantum interaction, while conscious qualia, which are C-L (because all conscious representations are C-L), is parallel to what is observed, classically, in measuring instruments as effects of this interaction. RWR-type interpretations preclude any ontology of the *physical* reality responsible for quantum phenomena, for example, von Neumann’s unitary ontology, which grounds most ontological views in QM.^11^ Some Q-L approaches to consciousness assume a mental ontology [e.g., (D’Ariano and Faggin, 2021)]. As noted, however, assuming an ontology of mental reality is very different from assuming that of physical reality. Physical reality is something that is assumed here to exist independently of us. Mental reality only exists in our experience, unless one assumes, on Platonist lines, the existence an independent ideal reality, which such Q-L approaches generally do not. Nor does the present view, although it assumes that the ultimate nature of mental reality, unique to each human subject, is of the RWR/IWI type. This assumption precludes a representation or even conception, and hence a mental ontology, of this reality.

Considering the unconscious is uncommon in mathematical Q-L modeling. One exception is Khrennikov’s argument (Khrennikov, 2015, 2021a), in which consciousness is viewed in terms of measurements performed on the unconscious, as decisions made by consciousness. It is not clear whether Khrennikov assumes a mental ontology, for example, based on von-Neumann unitary evolution. One might conjecture that he does, given his use of Ozawa’s argument concerning quantum measurement, which assumes this ontology (Ozawa, 1984, 1997; Khrennikov, 2021a). It is possible, however, that Khrennikov leaves this aspect of the situation open. A human subject, at least part of it, is thus also an interior measuring instrument which produces each such outcome. This is a plausible argument, considered by the present author in detail in Plotnitsky (2021b). I shall expand on it here by suggesting that human thinking, as Q-L in nature, creates, by performing something akin to a *quantum* measurement or (given the present view) rather observations, *both* conscious and unconscious representations, which are classical, but the efficacity of which is of the RWR/IWI type. If one adopts this view, the ultimate nature of the unconscious becomes stratified by Q-L reality, assumed here to an RWR/IWI-type reality, and C-like reality, assumed to allow for a classical realist representation.

This view thus extends to the present understanding of quantum measurement or, in the first place, observation to consciousness and the unconscious, and their interactions. To reprise this understanding, a quantum observation, *does not observe or measure* any preexisting property of the reality responsible of what is observed, but instead *establishes* quantum phenomena by an *interaction* between the instrument and this reality. Then what is so observed can be measured classically. In the case of consciousness, the outcomes of our observations upon the unconscious are manifested to our consciousness in classical representations and the corresponding language, through which these outcomes can then be made manifested to an outside observer. Not so in the case of the unconscious, where, however, the same type of observational process is still operative, creating classical representations, as manifested in dreams. It is a complex question whether our consciousness experiences dreams as such, although can have has a memory of a dream. Dreams do suggest, however, that our unconscious, too, contains classical representations. As discussed earlier, there are no analog of quantum objects identical to each other within the same type, such as electrons, in human thinking. Every instant of thought creates a new (“object” of) experience, responsible for the corresponding representations.

It is, thus, possible to see consciousness primarily as a mechanism that brings representations created by the unconscious into consciousness. Consciousness certainly has this function, perhaps as its primary function, with the unconscious as the dominant thinking agency. It is also possible, however, to take a more stratified view of both conscious and unconscious and of “measurement” there rather than only seeing consciousness as performing measurements/observations on the unconscious. In this view, unconscious thinking, too, involves, within itself, observations, with only some of them transferred to classical-like effects in consciousness, while others remain unconscious, possibly without ever becoming conscious, even in dreams.

This understanding is close to that of Freud, except that it is underlain by the RWR/IWI view of the ultimate reality responsible for all effects just noted. Freud’s view was, again, ontological. Both consciousness and the unconscious were seen as representable. On the other hand, while the RWR/IWI view in general allows one to assume that the ultimate reality defining thinking is unconscious, the present view assumes, along the lines just sketched, that the unconscious also contains classical representations created by this reality. Consciousness, again, contains both purely conscious representations and those transmitted from the unconscious. The ultimate RWR/IWI reality responsible for any such representations as effects is unconscious, if one assumes that consciousness is comprised of classical representations, some of which are created by the unconscious and others transmitted from the unconscious as already created there. This RWR-type reality may still ultimately be material, while all mental reality classical, which is, again, possible without adopting a physicalist view.

## “Three quarks for Muster Mark”: Qualia, decisions, and QFT-L literary models

Imagine somebody walking, with a map, along labyrinthine streets of a city with a great architecture (Venice is a good example, with its canals complicating one’s navigation even with a map). One can plan a trajectory ahead, but because nice buildings on other streets one passes by, or for no apparent reasons, one changes one’s route and makes new plans, consciously or unconsciously. One could model some such trajectories mathematically, say, by graph theory, and use computers do so, as the tour guide publishers probably do now, and thus model some of these decisions statistically, but only a small portion of them. The person on a walk deals with a much more multi-trajectorial and highly individualized field of decisions. T. S. Eliot captured this aspect of our life in his famous poem:

In a minute there is time

For decisions and revisions which a minute will reverse.

(“The Love Song of J. Alfred Prufrock,” 1915, ll.47–48)

Eliot’s “Do I dare?” is also a decision question, and he repeats it and “Do I dare or do I not dare?” in the poem several times. Eliot, including in this poem, undoubtedly made and reversed his decisions many times in choosing many words, reflecting qualia.

These considerations suggests that our thinking and representations it creates are akin to the way particles, viewed in this article as manifested only in the corresponding representations in observations, transform into one another in QFT, before an observation finalizes one of them or a set of them, new each time. I am not thus implying that “words” or even letters are akin to particles. As discussed below, the workings of language disallows assuming any fully stable self-identical linguistic “atomic” entities, such as words or even phonemes. The situation in QFT is contained by classification and mathematical handling, for example, in terms of symmetry groups, associated with elementary particles. Nevertheless, it was a remarkable feature of high-energy quantum physics that emerged with Dirac’s discovery of the positron as, according to Heisenberg, “perhaps the biggest change of all the big changes in physics of our [twentieth] century. It was a discovery of utmost importance because it changed our whole picture of matter” (Heisenberg, 1989, pp. 31). As noted earlier, in one and the same experiment in quantum electrodynamics, QED, after the initial preparation, say, of an electron, one finds in the next measurement in the corresponding region, not only an electron (or nothing), as in QM regimes, but possibly a positron, a photon, or an electron–positron pair; that is, in RWR-type interpretations, the phenomena (observed in instruments) that we associate with such entities. If one still speaks in terms of particles as existing independently, instead of a particle’s “motion,” one encounters a continuous birth and disappearances of particles, mathematically handled by the so-called creation and annihilation operators. In the present interpretation, the concept of a particle is an idealization applicable only at the time of observation. QED predicts which among such events can occur and with what probability. Once one moves to higher energies, the panoply of possible outcomes becomes still greater. In the case of QED, one only deals with electrons, positrons, and photons; in QFT, depending how high the energy is, one can find any elementary particle or combination of them.

As is well known, M. Gell-Mann borrowed the term “quark” from Joyce’s *Finnegans Wake* (Joyce, 2012, p. 118). The case, according to Gell-Mann’s own account, is more complicated:

In 1963, when I assigned the name “quark” to the fundamental constituents of the nucleon, I had the sound first, without the spelling, which could have been “kwork.” Then, in one of my occasional perusals of *Finnegans Wake* by James Joyce, I came across the word “quark” in the phrase “Three quarks for Muster Mark.” Since “quark” (meaning, for one thing, the cry of a gull) was clearly intended to rhyme with “mark,” as well as “bark” and other such words, I had to find an excuse to pronounce it “kwork.” But the book represents the dream of a publican names Humphrey Chimpden Earwicker. Words in the text are typically drawn from several sources at once, like the “portmanteau words” in [L. Carroll’s] *Through the Looking Glass.* From time to time, phrases occur in the book that are partially determined by calls for drinks at the bar. I argued, therefore, that perhaps one of the multiple sources of the cry “Three quarks for Muster Mark” might be “Three quarts for Muster Mark,” in which case the pronunciation “kwork” would be totally unjustified. In any case, the number three fitted perfectly the way quarks occur in nature (Gell-Mann, 1994, pp. 180–181).

Thus, the process of Gell-Mann’s naming quarks “quarks” was more intricate than merely borrowing the term from Joyce. The name was the product of a sequence of decisions, unlikely to be modeled mathematically, in contrast to the effects defining quarks in QFT. Where did the sound (possible spelled like “kwork”) come from? The name quark (just as “color” and “flavor” of quarks) is arbitrary and doesn’t reflect qualia as in the case of “electron” or “photons,” or “gluons,” which carry strong force binding, “gluing,” quarks inside the nuclei (We now know that there are six quarks).

The name quark does not appear to have any connection with particle transformation into each other in QFT. On the other hand, Joyce might have had this transformation in mind in writing his 1939 novel, which was influenced by quantum theory, conceivably by the discovery of antimatter, the first case on the QFT multiplicity. The subject was widely discussed at the time, just as the Higgs boson or black holes are now, and it was known to Joyce, as some passages in the novel suggest. While, however, the connections to quantum physics have been commented upon by scholars, those to QFT have not, to my knowledge, been considered. Joyce says in the novel, undoubtedly referring to his own project in the book: “I am working out a quantum theory about it for it is really most tantumising [a play on tantalizing] state of affairs” (Joyce, 2012, p. 149). Just as particles do in high-energy quantum physics, in Joyce’s novel words transform into each other, and new words are created, such as, famously, “chaosmos,” equally applicable to his novel and quantum physics (Joyce, 2012, p. 181). “Quarts” becoming “quarks,” surmised by Gell-Mann, might have been an example of such a transformation, still relating them phonologically. The analogy with QFT is, however, also about the birth of something new, more akin to the appearance of kwork as a sound to Gell-Mann, but equally transforming the signifiers and signified, or a referent (This follows F. de Saussure’s scheme in which the signified is the concept associated with a material sound or a written mark, making a sign, and the referent is what this sign refers to).

Joyce’s invocation of quantum theory is inserted into an elaboration that stages a sample of such transformations, associated with an overtly single name or signifier: “Talis.” A passage also parodies a scholarly text, intermixing this parody with the transformational play that a scholar, for example, a Joycean, would try to analyze. The text says, before moving to the Talis paragraph:

To put it all the more plumbsily. The speech form is mere surrogate. Whilst the quantity and tality (I shall explex what you ought to mean by this which its proper when and where and why and how in the subsequent sentence) are alternativomentally harrogate and arrogate, as the gates may be.
Talis is a word often abused by many passim (I am working out a quantum theory about it because it is really the most tantumizing state of affairs). A passim may frequent you to say: Have you been seeing much of Talis and Talis those times (Joyce, 2012, pp. 149–150).

The quality and tality, which may also designate that which precedes “totality,” “alternativomentally harrogate and arrogate, as the gates may be” is what thinking, including in language, does. This is the most tantumising/tantalizing state of affairs that requires a Q-L theory, more akin to QFT, even though, while similar epistemologically, in terms of the key postulates (QI, QD, QP/QS, and no cloning) used, this quantum theory is no longer mathematizable. The word “talis” means, fittingly, “such,” “of such sort,” or “such as,” in Latin. It might appear that “talis” is abused by many *passims* because “it is used without a clear referent or as a substitute for rigorous thinking” (Ku, 2021, p. 143).^12^ The opposite is more likely to be true, especially with QFT in mind. The rigorous thinking consisted in realizing that a (single) clear referent or even a signified is no longer possible. What is an electron? It is such and such thing, a “talis,” that has such and such effects, some of which, but only some (such as mass, charge, or spin), are the same. The reality responsible for them is, however, never the same in actual experiments, even if, paradoxically (if one thinks classically), there is no way to ever distinguish two electrons as such. Consider, the following passage, a “broadcast” containing multiple allusions to quantum physics:

*The abnihilisation of the etym by the grisning of the grosning of the grinder of the grunder of the first lord of Hurtreford expolodotonates through Parsuralia with an ivanmorinthorrorumble fragoromboassity amidwhiches general uttermosts confussion are perceivable moletons skaping with mulicules while coventry plumpkins fairlygosmotherthemselves in the Landaunelegants of Pinkadindy. Similar scenatas are projectilised from Hullulullu, Bawlawayo, empyreal Raum and mordern Atems.* (Joyce, 2012, p. 353; italics in original).

*“The abnihilisation of the etym*” refers to both atoms and words (etymology). One can read this phrase as alluding, to E. Rutherford’s experiments, the work for which he eventually received a knighthood, thus becoming a lord. His experiments showed that regular (chemically defined) atoms are no longer indivisible Democritean atoms. The signifier Rutherford is itself “atomized” and recomposed as the first lord Hurtreford, and then relate this passage to QM (Ku, 2021, p. 137). Limiting oneself to reading Joyce’s literary “quantum theory” to these connections or even those to QM, misses Joyce’s literary “QFT,” as a more radical form of transformational play enacted by his text. By the time of *Finnegans Wake*, elementary particles became new atoms of nature, transforming into each other in QFT regimes, and phonemes new atoms of language in linguistics. These concepts no longer allowed one to maintain any particle or linguistic identity, apart from the moment when an experiment or reading, or thought, fixes it. Taking advantage of Latin “elementa” as designating both, Lucretius famously compared, undoubtedly with his own composition of *De Rerum Natura* of words in mind, atoms to the letter of the alphabet (Lucretius, 2009, 2. ll. 688–689). This joint, physical and linguistic, atomism was clearly on Joyce’s mind. His text, however, not only contains more complex and longer “molecular” chains (with some becoming, as it were, “alive,” and begetting other chains, which multiply in turn), but also preclude one from fixing the identity of word or phonemes, transformed by a reading. A reading could of course also transform the “atoms” of Lucretius’s or any text. However, Joyce’s text:

(1) Enacts such transformational effects, by means of a hitherto unprecedented QFT-like signifying transformational play, as passages cited here exemplify;

(2) By doing so, *enacts* what it describes, a phenomenon known as dedoublement;

(3) Theorizes this play, by making a literary QFT-L theory about it; and

(4) Finally, ironically parodies a scholarly text, which is another dedoublement.

Some of these aspects of the novel has been noted by commentators, including in connection to quantum theory. I would argue, however, that, contrary to a common view (which follows realist thinking, moreover, dealing with QM and not QFT) this is not a matter of a textual mimesis of “the quantum world,” as different from the classical world. In the RWR view, there can be no *mimesis* of “the quantum world,” because the ultimate reality responsible for quantum phenomena is beyond representation or even conception. There are only transforming effects, which relate to that which is beyond thought, at least in physics, and as I suggest here, possibly to the same (IWI) form of mental reality. According to Bohr, there is no such as thing as a “quantum world,” but only probabilistic predictions of effects of the RWR-type reality, which are beyond any conception (including as a “world”), on the classical world we observe (Plotnitsky, 2021c, p. 174). It is possible that Joyce himself assumed a more realist view of this situation either in quantum theory or of human thinking and language. The text, however, allows for the RWR/IWI-type and QFT-L reading. One might argue that, while *Ulysses* (1922) relates to relativity and the old quantum theory (before QM), along more realist lines, *Finnegans Wake* does so in an RWR/IWI type way, *via* QFT. It is also possible that Gödel’s incompleteness theorems in mathematics, discovered in 1931 and much discussed at the time, played a role in *Finnegans Wake*. These theorems radically changed our view of mathematical reality (Plotnitsky, 2019a, pp. 202–208; Plotnitsky, 2020b, pp. 21-25).^13^

One can never fully account for or always predict such transformations, either those by Joyce in creating them or by a reader reading them, in contrast to QFT which, by only dealing with Shannon information, one can probabilistically predict the particles’ transformations regardless of a particular observing subject. The semantic information potentially brought in by such a subject can be disregarded in physics and sometimes in Q-L modeling. In human thinking and in in Joyce’s novel, as a literary enactment of thinking, this is no longer possible, which limits the use Q-L, even QFT-L, *mathematical* models. With this last qualification in mind, however, these transformations are, and might have been thought of by Joyce as, QFT-L. They are defined by the creation and annihilation of linguistic units, to the degree that one can have such linguistic units. In fact, the word “annihilation” emerged in physics in the context of QFT, which is, hence, a more likely reference here. Rutherford’s experiments did not annihilate atoms, but decomposed them into particles, assumed by him as retaining their identity in each experiment, as still possible in QM. This stability was no longer possible in high-energy physics and QFT. This new understating of the workings of nature would have appealed to Joyce and might have shaped linguistic transformations enacted by *Finnegans Wake*. Nor can the multitude of trajectories defined by the passages here considered or most others in the novel, be contained by a reading. Any such trajectory can be unpredictably transformed and new ones created by other readings, beyond anything Joyce might have thought. The no-cloning postulate applies here. Every reading of a text, or every (re)construction of the author’s experience that creates it, make it unique to each reader, just as creating his text was unique to Joyce.

My main concern is, however, not the impact of quantum theory on *Finnegans Wake*, helpful as it might have been in making the novel what it is, an impact that is found elsewhere in modernist literature, as in S. Beckett or R. Musil, or later T. Pynchon (e.g., Plotnitsky, 2020a,2021d). I am more concerned with what Joyce’s novel or other literary works do for our understanding of quantum theory and its relation to the nature of human thinking, specifically as concerns Q-L modeling, in particular, how likely and to what degree even QFT-L *mathematical* models are to handle human thinking, which are QFT-L/RWR *epistemologically*. The thinking that led Joyce to what became, in the present reading, an RWR-type enactment of human thinking known as *Finnegans Wake* reflects the fundamental nature of these limitations. More crucially, the RWR-type epistemology of all Q-L models suggests a new way of *thinking* about human thinking and language, even though mathematical Q-L models cannot capture their richness.^14^

Proust gave the hard problem a powerful literary expression or staging in numerous passages, which also reflect on it. Thus:

[T]hings—and among them a book in a red binding—as soon as we have perceived them are transformed within us into something immaterial, something of the same nature as our preoccupations and sensations at that particular time, with which, indissolubly, they blend. A name read long ago in a book contains within its syllables the strong wind and the brilliant sunshine that prevailed while we were reading it. … Yet it is precisely this essence that an art worthy of the name must seek to express; then at least, if it fails, there is a lesson from its impotence (whereas from the successes of realism [that is not concerned with the inner experience] there is nothing to be learned), the lesson that this essence is, in part, subjective and incommunicable (Proust, 2003, v. 6, p. 285).

It is so for the most part. This passage tells us that our inner experience, expressly conveyed here, for example, by the red of the book binding, and throughout his novels, would be impossible to represent mathematically, or predict by any means. The same is true as concern our reading of this passage, which is also about reading and our reading of it. There are far too many qualia in every passage of Proust or in human experience, for example, “the strong wind and the brilliant sunshine” one can experience in minute, during which “there is time for decisions and revisions that a minute will reverse.” Proust, certainly, did in writing his novel, just as did Joyce, who gave this process a QFT-like version.

What is, then, shared by quantum physics and Q-L models of human thinking is their Q-L ontological and epistemological architecture, here interpreted in RWR/IWI terms. This architecture is defined by the uniqueness of the state of either nature or thought at any given point in time by the QI postulate, leading to the no cloning postulate and coupled to the ultimate discreteness of each by the QD postulate, even though our conscious experience of these events may appear as a continuous flow. How nature and thought ultimately work may be unknowable or even inconceivable. The QP/QS postulate is a consequence, except that we may not have a mathematical theory to use it. Freud’s German word for the unconscious was *das Unbewusste* [unknowable] originally used by F. Schelling in the eighteenth century in response to Kant. It is unknowable even to ourselves, in contrast to our conscious thought, only unknowable to others. The unthinkable, *das Undenkbare*? Some poets, Schelling’s contemporaries, such as H. von Kleist and F. Hölderlin, thought that this might be the case (Plotnitsky, 2021d).

Freud adopts Kant’s view based on the difference between phenomena, defined by appearances or *representations* in our mind, which are available to our knowledge, vs. noumena or things-in-themselves as they actually exist, which are beyond our knowledge, but not necessarily beyond thought, as they would be in the strong RWR view. Freud also builds on Kant’s view that things-in-themselves, noumena, may be mental. In Freud this noumenal domain is the unconscious. In spite, however, of terming the unconscious *das Unbewusste*, Freud was more optimistic than Kant. In his 1915 “The Unconscious,” Freud says, perhaps also thinking of quantum theory then, such as Bohr’s 1913 theory: “The mental, like the physical, is not necessarily in reality what it appears to us to be. It is, however, satisfactory to find that the correction of inner perception does not present difficulty so great as outer perception—that the inner object is less hard to discern truly that in the outside word” (Freud, 1969b, p. 121).

Freud might have been overconfident as concerns the capacity of his theory to discern the ultimate nature of this “inner object.” This article suggests, on RWR/IWI lines, that we may not have such a capacity, as concerns the ultimate nature of the reality, because, in the present view, part of the unconscious contains classical-like archive of representations, stratifying the unconscious into the classical and RWR/IWI strata. As noted, conscious and unconscious representations are still different as classical-like effects of a mental RWR/IWI type reality. Unconscious ones remain hidden and could only be manifested, often indirectly, in conscious representations, for example, *via* dreams or memories of dreams, or long-term memory. This makes conscious representations arising from the unconscious double effects of the ultimate RWR/IWI-type reality: they are representations of representations, representations of unknown (but not necessarily unknowable) representations. Maintaining the parallel with observation in quantum physics, *as a creation of new phenomena*, both conscious *and* unconscious (classical) representations might be seen as classical effects of Q-L observations. By the same token, quantum-to-classical information transfers may also be seen as observations, again, extended to the unconscious.

It is crucial (hence my emphasis above) that in the present view an observation is a creation of phenomena by means of the interactions between one or another agency (such as measuring instruments or consciousness) with the ultimate reality considered (such as certain strata of matter or the unconscious) rather than observing or measuring the preexisting or even contemporaneous properties of this reality. One might see this reality as that of the *mental* things-in-themselves, which Kant invoked nearly three centuries ago, but are conceived of here more radically, along (strong) RWR and IWI lines, as beyond conception. Heisenberg saw Kant’s things-in-themselves, if one assumes them at all in physics, as “mathematical structure[s]” (Heisenberg, 1962, p. 52). As noted, for Heisenberg, these mathematical structures were representing the ultimate nature of physical reality apart from conventional physical concepts, such as those of classical physics or relativity. This view implies a form of mathematical ontology, although in this case as representing the ultimate nature of physical reality, rather than the ultimate nature of human thinking. As beyond thought, the ultimate constitution of the reality responsible for thinking cannot be observed either, any more than that of the material reality responsible for quantum phenomena could be in the strong RWR view. IWI reality thus suggested is a product of the material reality defined by the workings of our neurological machinery, whether the latter is described in a realist, such as classical, or RWR (possibly quantum) way. This would not affect the present argument, insofar as the hard problem remains beyond a functional solution, because the *emergence* of consciousness or thinking from this neurological reality would still be governed by the RWR view.^15^

That, again, does not mean that scientific, including mathematical, approaches, such as Q-L ones, to consciousness and thinking, including the unconscious are not possible. I only argue for the limited range of mathematical models, Q-L or C-L, in considering human thinking, especially if used without, rather than in cooperation with non-mathematical models, scientific or philosophical. Moreover, I argue that these approaches, or quantum theory in the first place, suggest different ways in which we can relate to the world, the world of nature and the world of thought, in their irreducible interaction.

There is an additional possibility, which leads to a different type of models or theories, conforming to the QI, no-cloning, QD, and QP (but not QS) postulates, and RWR views, which I would like to suggest in closing, extending and revising the argument offered in Plotnitsky (2017). I shall call such models or theories singularized probabilistic (SP) models or theories, keeping in mind their RWR-type character (Realist SP models are possible, too, but are put aside here). Such models can only be briefly sketched here, but this will suffice for explaining their possibility.

I recall first that, as reflected in complementarity, in QM or QFT, there is no uniform physical law applicable to quantum phenomena in all contexts, while the same mathematical formalism of QM or QFT works in all contexts. Depending on whether an interpretation is statistical or Bayesian probabilistic (there are, again, various versions of each), individual quantum events are either assumed to be random or to be subject to the probabilistic laws. The application of one or another of these laws is established by the context of observation, which defines the initial conditions, understood here as a creation of the corresponding quantum phenomena, rather than observing preexisting properties of quantum objects. By contrast, an SP-model or theory arises not only because each individual case is defined by its own singular law, but also because this law is encoded in a different mathematical model applicable only in this case. It does not conform to a contextual probabilistic or statistical law, from a (determinable) set of such laws defined by the theory, which uses a single mathematical model, as in the case of QM or QFT. One makes a Bayesian bet on of *the model itself* developed on the basis of the information (a Bayesian prior) one has, information defining the initial conditions.

It may be strange to think of having a separate theory for each event, but in practice in economics or finance, for example, this is, *in effect*, not so uncommon. Economists use different mathematical models, only some which will make a good prediction in a given case. Hence, while it may be assumed to be a general model, such a model will function in practice as an SP-model, insofar as a different model or set of models will be adopted for the next case. Nevertheless, the concept of an SP model or theory is a radical idea, which is to my knowledge rarely, if ever, entertained, in science. While speaking of the unreasonable effectiveness of mathematics in natural sciences, Wigner also noted its essential limit there: “We have seen that there are regularities in the events in the world around us which can be formulated in terms of mathematical concepts with an uncanny accuracy. There are, on the other hand, aspects of the world concerning which we do not believe in the existence of any accurate regularities. We call these initial conditions” (Wigner, 1960, p. 14). In the case of an SP model, one deals with a possible (and possibly quick) change of initial conditions.

It is not clear to what extent SP theories and, especially, mathematical models are scientifically viable. For an effective scientific practice to be possible, one might need regularities beyond those found in each singular situation, for which a mathematical model, unique to it, would be introduced. Such changes of laws and models could, in principle, be governed mathematically: one could have a set of models mathematically parameterized to allow one to use them for different individual situations and to adjust them to make effective predictions. That would, however, in essence amount to a single, if complex, mathematical model, and not an SP one.

Would mathematical-experimental sciences, as they are practiced now, still be possible, if only SP models could be used? Furthermore, there might, in some domains, be individual cases the character of which will defeat our attempt to treat them by mathematical means, as often happens, as I have argued here, in human thinking and decision making. One would need, then, to use both mathematical, either the more standard or the SP type (both may be C-L or Q-L), and non-mathematical, such as qualitative, theories and models, including epistemologically Q-L. One would be dealing with a repertoire of models, only some of which are mathematical, possibly both of C-L and Q-L, and hence possible RWR/IWI types, and a form of model engineering, as it were.^16^ Could this situation also emerge in physics, for example, in dealing with quantum gravity? This is not inconceivable. If it does, it may not, and is, I would surmise, unlikely to, end the mathematical-experimental character of, and hence mathematical modeling, in physics, a character which have defined it from Galileo on. It will, however, require bringing into physics other ways of thinking, similarly to psychology or economics.

Apart from life itself, where it is ubiquitous, the radical singularity of events or sequences of events is, as we have seen, more familiar in literature. Literature is often and, arguably, primarily concerned with the particular or the singular, for example, with a unique life history of a novel’s protagonist, even in so-called “realist” literature. Whatever else it may portray, such as culture or politics, to remain literature deserving the name, realist literature must, as Proust says, be primarily about a representation or the unrepresentable nature of human experience as inner experience, and the relationships between such inner experiences. Many works of poetry could serve as immediate examples. But the whole history of the novel, too, from M. Cervantes to L. Tolstoy and, especially, F. Dostoyevsky and beyond would represent this literature of inner experience, something that they in fact share with poetry, even though all these authors deal with many other things in their works. In modernist literature, however, as in T. S. Eliot, Woolf, Joyce, Beckett, or Musil, in part under the impact of quantum physics, dealing with inner experience acquired, epistemologically Q-like features.

This literature also grounded them in RWR/IWI-type epistemology, at least in certain readings of these works. But then, as stated from the outset, any RWR/IWI type interpretation is only assumed here to be an interpretation, one among other possible interpretations, of quantum theory or thinking, rather than as defining the true nature of reality. Nevertheless, it emerged from confronting “really the most tantumazing state of affairs,” which requires one to “work out quantum theory about it,” physical, philosophical, literary or other. Perhaps Joyce said “really” because this state of affairs requires a “quantum theory” of what is *real*, even if its ultimate nature of this real is beyond this or any possible theory, or beyond thought. Thought, however, can still relate to this real, through effects it has on the world and, as part of it, on thought itself. To be able to do so is an achievement of thought.

## Data availability statement

The original contributions presented in this study are included in the article/supplementary material, further inquiries can be directed to the corresponding author.

## Author contributions

The author confirms being the sole contributor of this work and has approved it for publication.

## Footnotes

1 This article in part builds on the argument offered in Plotnitsky (2021b), which, however, does not consider the question of the limitations of mathematical Q-L models. There is some overlapping, primarily in the definitions of concepts of quantum theory, definitions still revised here.

2 There is a reported remark by I. M. Gelfand on “the unreasonable ineffectiveness of mathematics in biology,” which even led to the Wikipedia article on the effectiveness or ineffectiveness of mathematics in various scientific fields (e.g., Velupillai, 2005). My claim only concerns cognitive and social sciences, and I do not find the limited effectiveness of mathematics there unreasonable.

3 The probability density function specifies the probability of the random falling *within a particular range of values*, as opposed to taking on a single value. This probability is given by the integral of this variable’s probability density function over this range. The probability density function is non-negative, and its integral over the entire space is equal to 1.

4 Quantum phenomena are assumed here to be defined by the fact that in considering them the Planck constant, *h*, must be taken into account, putting aside qualifications of this definition, necessary in general but not germane for this article (Plotnitsky, 2021c, pp. 37–38). See, however Khrennikov (2021b).

5 In considering scientific theories, the concept just outlined is often called “scientific realism.” This outline would, however, apply to most forms of realism in science or philosophy that I am familiar with, although not *all* possible form of it, which would be impossible. I shall also refer to realist theories as ontological. Although the terms “realist” and “ontological” sometimes designate different concepts, they are close and will be used interchangeably here. Another common term for realist theories is “ontic.”

6 These experiments have been considered from the RWR perspective by the present author in Plotnitsky (2016, 2021c).

7 In this respect, the present view is close to, although not the same as that of quantum Bayesianism or QBism (e.g., Fuchs et al., 2014). There are different versions of the Bayesian view. Most generally, it defines probability as a (quantified) degree of belief concerning a possible occurrence of an individual event based the relevant information one possesses, defined by a prior probability distribution, or the prior, of an uncertain quantity, which expresses one’s beliefs concerning this quantity before new evidence is taken into account. This evidence may change one’s degree of belief, “updating” the prior, by using the so-called Bayes theorem. This new estimate is called the posterior probability. This makes one’s probabilistic estimates, in general, subjective, although there may be agreement concerning them (The Bayes theorem is more general and applies to the frequentist view as well). The frequentist view defines probability in terms of sample data by emphasis on the frequency or proportion of these data, which is considered more objective. The absence, in general, of exact predictions in dealing with individual events in quantum physics may be interpreted on either Bayesian or frequentist lines (e.g., Plotnitsky, 2016, pp. 173–186).

8 That need not, and in RWR-type interpretations does not, mean to this non-commutativity *represents* this situation; it is only part of the formalism of QM that predicts the outcomes of the experiments defined by this situation (Plotnitsky, 2021c, pp. 119–122).

9 Several authors are credited with the proof (Park, 1970; Dieks, 1982; Wootters and Zurek, 1982).

10 I put aside controversies surrounding the hard problem, the existence of which has been disputed. I don’t take a strong position on its existence, although I am inclined to agree with Chalmers. The idea of the hard problem is, in any event, useful for my argument.

11 A rare exception is M. D’Ariano and F. Faggin’s ontology based on the non-unitary quantum evolution and opposed by them to von Neumann’s unitary evolution (D’Ariano, 2020; D’Ariano and Faggin, 2021). I discussed their approach in Plotnitsky (2021b).

12 The article just cited assumes a realist perspective, with gestures toward Bohmian theory and many worlds interpretation (obviously, not known to Joyce, who died in 1941) and contains misleading statements concerning Bohr and, uncritically identifying them, the Copenhagen interpretation. Thus: “Bohr’s (in)famous (and mainstream) Copenhagen Interpretation contends that it is the intervention of measurement that collapses quantum superposition and produces the determining effect” (Ku, 2021, p. 139). As explained, in Bohr’s view, quantum superposition is not anything independently physical with which a measurement intervenes, “collapsing” it, but part of the mathematical machinery that enables one to predict the probabilities or statistics of measurements.

13 The first incompleteness theorem states that any consistent formal system (that contains arithmetic of natural numbers), is incomplete: there are statements of the language of this system that are undecidable, that is, can neither be proved nor disproved in this system. Gödel’s second theorem states that the proposition that such a system itself is consistent cannot be proven, is an undecidable proposition. The second theorem allows that such a system could be shown to be inconsistent.

14 Language is rarely considered in this connection. See, however, an intriguing argument offered in Bruza et al. (2009) and a general argument (from a realist perspective) in Aerts and Beltran (2022). The latter article sees “words as quanta of language,” adding, wisely, a question mark to its title. While, as the article shows *via* quantitative analysis, this hypothesis is workable in certain settings (even in certain simple literary narratives), it is difficult to reconcile with the view of language suggested by Joyce’s novels. As I argue here, these novels are *enactments* of the workings of language as something cannot be “atomized” into words, if at all.

15 In dealing with Q-L modeling of thinking (even if one does not assume that they arise from the quantum physics of the brain), one still deals with open biological systems. This may mean that one needs to always use the formalism of open quantum systems, whereas in quantum theory where considering an isolated system can be made precise (even if it is still an approximation) and suffices in most cases. This argument follows that of Schrödinger, for all biological systems, in *What is Life?* (Schrödinger, 1944). For an informative discussion, see Basieva et al. (2021). The subject, which received more attention in recent Q-L approaches, would require a separate analysis, but the epistemological Q-L argument of this article would apply in this case, assuming that one adopts an RWR-type interpretation, equally allowed by isolated and open quantum systems. I might add that inner experience, while always *inner* and hence consistent with the hard problem, essentially depends on our interactions (material, mental, or social) with the world and would not be possible otherwise. It is an open system, while preserving its inner aspects.

16 This type of practice is similar to what C. Lévi-Strauss advocated as “bricolage,” and he used certain algebraic (group-theoretical) models as part of bricolage. He juxtaposed a bricoleur to an engineer, who would, conventionally, use a more controlled form of modeling (Lévi-Strauss, 1966, p. 17). This opposition, as Derrida shows, is unsustainable as an unconditional opposition: a bricoleur is also an engineer and an engineer a bricoleur, although the balance of both can be different in each specific case (Derrida, 1978, pp. 285–288). Lévi-Strauss’s approach in not of RWR-type and bypasses the complexity of inner experience, which may require Q-L epistemological models and thus open the possibility of the RWR/IWI type approach (Plotnitsky, 2019b).

## References

[B1] Aerts D. Beltran L. (2022). Are words the quanta of human language? Extending the domain of quantum cognition. *Entropy* 24:6. 10.3390/e24010006 35052032 PMC8775064

[B2] Basieva I. Ozawa M. Khrennikov A. (2021). Quantum-like modeling in biology with open quantum systems and instruments. *Biosystems* 201:104328. 10.1016/j.biosystems.2020.104328 33347968

[B3] Bohr N. (1935). Can quantum-mechanical description of physical reality be considered complete? *Phys. Rev.* 48 696–702. 10.1103/PhysRev.48.696

[B4] Bohr N. (1987). *The Philosophical Writings of Niels Bohr.* Woodbridge, CT: Ox Bow Press.

[B5] Bruza P. Kitto K. Nelson D. McEvoy C. (2009). Is there something quantum-like about the human mental lexicon? *J. Math. Psychol.* 53 362–377. 10.1016/j.jmp.2009.04.004 20224806 PMC2834425

[B6] Busemeyer J. Bruza P. D. (2014). *Quantum Models of Cognition and Decision.* Cambridge: Cambridge University Press.

[B7] Chalmers D. (2010). *The Character of Consciousness.* Oxford: Oxford University Press. 10.1093/acprof:oso/9780195311105.001.0001

[B8] D’Ariano G. M. (2020). No purification ontology, no quantum paradoxes. *Found. Phys.* 50 1921–1933. 10.1007/s10701-020-00398-6

[B9] D’Ariano G. M. Faggin F. (2021). Hard problem and free will: an information-theoretical approach. *arXiv* [preprint]. *arXiv:2012.06580v2.

[B10] Derrida J. (1976). *Of Grammatology, Transl.* Baltimore, MD: John Hopkins University Press.

[B11] Derrida J. (1978). *Writing and Difference.* Chicago: University of Chicago Press.

[B12] Dick P. K. (2008). *Do Android Dream of Electric Sheep?.* New York, NY: Del Rey.

[B13] Dieks D. (1982). Communication by EPR devices. *Phys. Lett. A* 92 271–272. 10.1016/0375-9601(82)90084-6

[B14] Dirac P. A. M. (1958). *The Principles of Quantum Mechanics*, 4th Edn. Oxford: Clarendon, rpt.

[B15] Freud S. (1969a). *An Outline of Psychoanalysis, Transl.* New York: W. W. Norton.

[B16] Freud S. (1969b). *General Psychological Theory, Transl.* New York: W. W. Norton.

[B17] Frigg R. Hartmann S. (2012). “Models in science,” in *Stanford Encyclopedia of Philosophy*, ed. Zalta E. N..

[B18] Fuchs C. A. Mermin N. D. Schack R. (2014). An introduction to QBism with an application to the locality of quantum mechanics. *Am. J. Phys.* 82 749–754. 10.1119/1.4874855

[B19] Gell-Mann M. (1994). *The Quark and the Jaguar: Adventures in the Simple and the Complex.* New York: W. H. Freeman. 10.1063/1.2808634

[B20] Haven E. Khrennikov A. (2013). *Quantum Social Science.* Cambridge: Cambridge University Press. 10.1017/CBO9781139003261

[B21] Heisenberg W. (1925/1968). “Quantum-theoretical re-interpretation of kinematical and mechanical relations,” in *Sources of Quantum Mechanics*, ed. Van der Waerden B. L. (New York, NY: Dover), 261–277.

[B22] Heisenberg W. (1930). *The Physical Principles of the Quantum Theory.* New York: Dover, rpt.

[B23] Heisenberg W. (1962). *Physics and Philosophy: The Revolution in Modern Science.* New York: Harper & Row.

[B24] Heisenberg W. (1989). *Encounters With Einstein, and Other Essays on People, Places, and Particles.* Princeton: Princeton University Press.

[B25] Joyce J. (2012). *Finnegans Wake.* Oxford: Oxford University Press.

[B26] Kant I. (1997). *Critique of Pure Reason, Transl.* Cambridge: Cambridge University Press.

[B27] Khrennikov A. (2015). Quantum-like modeling of cognition. *Front. Phys.* 3:77. 10.3389/fphy.2015.00077

[B28] Khrennikov A. (2021a). Cooperative Functioning of Unconscious and Consciousness From Theory of Open Quantum Systems: www.preprints.org. MDPI, Basel, Switzerland. 10.20944/preprints202103.0454.v1

[B29] Khrennikov A. (2021b). Is the devil in h? *Entropy* 23 632. 10.3390/e23050632 34069443 PMC8159144

[B30] Ku P. (2021). “The abnihilisation of the etym”: Finnegans wake’s entanglement in quantum ideality. *Concent. Liter. Cult. Stud.* 47 129–148.

[B31] Ladyman J. (2016). “Structural realism,” in *Stanford Encyclopedia of Philosophy*, ed. Zalta E. N..

[B32] Lévi-Strauss C. (1966). *The Savage Mind.* Chicago: University of Chicago Press.

[B33] Lucretius T. C. (2009). *On the Nature of the Universe, Transl.* Oxford: Oxford University Press.

[B34] Mehra J. Rechenberg H. (2001). *The Historical Development of Quantum Theory.* Berlin: Springer.

[B35] Ozawa M. (1984). Quantum measuring processes for continuous observables. *J. Math. Phys.* 25 79–87. 10.1063/1.526000

[B36] Ozawa M. (1997). An operational approach to quantum state reduction. *Ann. Phys.* 259 121–137. 10.1006/aphy.1997.5706

[B37] Park J. (1970). The concept of transition in quantum mechanics. *Found. Phys.* 1 23–33. 10.1007/BF00708652

[B38] Penrose R. (1995). *The Emperor’s New Mind.* Oxford: Oxford University Press.

[B39] Plotnitsky A. (2016). *The Principles of Quantum Theory, from Planck’s Quanta to the Higgs Boson: The Nature of Quantum Reality and the Spirit of Copenhagen.* New York: Springer/Nature. 10.1007/978-3-319-32068-7

[B40] Plotnitsky A. (2017). The real and the mathematical in quantum modeling: From principles to models and from models to principles. *Front. Phys.* 5:19. 10.3389/fphy.2017.00019

[B41] Plotnitsky A. (2019a). “On the concept of curve: Geometry and algebra, from mathematical modernity to mathematical modernism,” in *Geometry in History*, eds Dani S. G. Papadopoulos A. (Berlin: Springer/Nature), 153–212. 10.1007/978-3-030-13609-3_5

[B42] Plotnitsky A. (2019b). Structure, sign, and play and the discourse of the natural sciences: After the hyppolite-Derrida exchange. *Mod. Lang. Notes* 134 253–266. 10.1353/mln.2019.0105 34409987

[B43] Plotnitsky A. (2020a). Demons of chance, angels of probability: Thomas Pynchon’s novels and the philosophy of chance and probability. *Trans. Am. Stud. J.* 1:15498. 10.4000/transatlantica.15498

[B44] Plotnitsky A. (2020b). “The ghost and the spirit of Pythagoras: The twentieth and twenty-first century mathematics between and beyond geometry and algebra,” in *Handbook in the History and Philosophy of Mathematics*, ed. Sriraman B. (Berlin: Springer/Nature). 10.1007/978-3-030-19071-2_7-1

[B45] Plotnitsky A. (2021a). Nature has no elementary particles and makes no measurements or predictions: Quantum measurement and quantum theory, from Bohr to Bell and from Bell to Bohr. *Entropy* 23:1197. 10.3390/e23091197 34573822 PMC8470679

[B46] Plotnitsky A. (2021b). “On decisions and revisions which a minute will reverse”: consciousness, the unconscious and mathematical modeling of thinking. *Entropy* 23:1026. 10.3390/e23081026 34441166 PMC8391280

[B47] Plotnitsky A. (2021c). *Reality Without Realism: Matter, Thought, and Technology of Quantum Physics.* Heidelberg: Springer/Nature. 10.1007/978-3-030-84578-0

[B48] Plotnitsky A. (2021d). “The paradoxical interplay of exactitude and indefiniteness: Reality, temporality, and probability, from Hölderlin to Heisenberg to Musil,” in *Physics and Literature: Concepts-Transfer-Aestheticization. Heydenreich A, Mecke K*, (Berlin: De Gruyter), 197–230. 10.1515/9783110481112-009

[B49] Proust M. (2003). *In Search of Lost Time.* New York: Modern Library.

[B50] Schrödinger E. (1944). *What is Life?.* Cambrdige: Cambridge University Press.

[B51] Tversky A. Kahneman D. (1973). Availability: A heuristic for judging frequency and probability. *Cogn. Psychol.* 5 207–237. 90033 10.1016/0010-0285(73)90033-9

[B52] Tversky A. Kahneman D. (1983). Extensional versus intuitive reasoning: The conjunction fallacy in probability judgment. *Psychol. Rev.* 90 293–315. 10.1037/0033-295X.90.4.293 PMC4658440 26635712

[B53] Velupillai K. V. (2005). Unreasonable ineffectiveness of mathematics. *Cambridge J. Econ.* 29 849–872. 10.1093/cje/bei084 33766624

[B54] Von Neumann J. (1932). *Mathematical Foundations of Quantum Mechanics.* Princeton: Princeton University Press.

[B55] Wigner E. P. (1960). The unreasonable effectiveness of mathematics in the natural science. *Comm. Pure Appl. Math.* 13 1–14. 10.1002/cpa.3160130102

[B56] Wootters W. Zurek W. (1982). A single quantum cannot be cloned. *Nature* 299 802–803. 10.1038/299802a0

